# Lectures on the Principles and Methods of Medical Observation and Research

**Published:** 1857-04

**Authors:** 


ART. VII.—*Lectures on the Principles and Methods of Medical Observation and Research. For the Use of Advanced Students and Junior Practitioners.* By THOMAS LAYCOCK, M. D., F. R. S. E., F. R. C. P., Professor of the Practice of Medicine, and of Clinical Medicine, in the University of Edinburgh, etc. Philadelphia: BLANCHARD & LEA. 1857.

This little volume of two hundred pages, consists of seven lectures, delivered in the course of Clinical Lectures at the Edinburgh School of Medicine.

We do not know that we can better define the objects of the work than by quoting directly from the volume, the following explanation of the circumstances which gave it origin:

"When about to enter for the first time upon his duties as Professor of Clinical Medicine, and to deliver the summer course of clinical lectures for 1856, in the University of Edinburgh, the author looked about for some elementary work on the inductive philosophy which he could recommend to his class, for their instruction and guidance in clinical observation and research. He found several sufficiently able manuals of *physical diagnosis*

adapted to students; and good elementary works on the uses of the microscope and on the *routine* of the clinical wards, with systematic instructions "how and what to observe." But he found none which instructs the medical student in a simple and easy form how to use his reason; none which explains to him in especial the nature of the mental processes by which knowledge is acquired in his particular sphere of labor; none which teaches him the applications to practical medicine of those aids to the intellectual powers which modern inductive philosophy uses so commonly and so efficiently. The student would inquire in vain for a short and practical exposition of the numerical method of research, in its special applications to practical medicine, or of that still more effective and philosophical method, the *analogical;* a method which, when once understood, is singularly easy of application, and equal (the writer is deeply convinced) to the solution of all the problems of life and organization that it is possible for the intellect of man to conceive, however protound they may be. A method, in short, of unlimited powers, and specially adapted to the needs of medical science."

Without attempting to decide whether the author has accomplished all that he aimed to perform, we can recommend the work to those for whom it was designed. It will be found a useful aid in the observation of disease, and a guide in setting a just estimate upon symptoms. Many of the sources of error in medical judgments are pointed out, and the close connection existing between theory and practice, is insisted upon. Those astute medical men, who are constantly harping upon their "practical knowledge," will, we apprehend, find some portions of this little volume less flattering to their assumptions of superior wisdom than they will be willing to admit. N.

